# Testing considerations for phosphatidylinositol‐3‐kinase catalytic subunit alpha as an emerging biomarker in advanced breast cancer

**DOI:** 10.1002/cam4.3278

**Published:** 2020-07-22

**Authors:** Deborah L. Toppmeyer, Michael F. Press

**Affiliations:** ^1^ Department of Medicine Robert Wood Johnson Medical School Rutgers Cancer Institute of New Jersey New Brunswick NJ USA; ^2^ Department of Pathology Keck School of Medicine University of Southern California Los Angeles CA USA

**Keywords:** advanced breast cancer, alpelisib, biomarker, companion diagnostic, *PIK3CA*

## Abstract

Breast cancer is the most common cancer in women, and approximately 71% of carcinomas are hormone receptor‐positive (HR+) and human epidermal growth factor receptor 2‐not‐amplified (HER2‐negative). Pathogenesis of breast cancer is associated with dysregulation of several signaling pathways, including the phosphatidylinositol‐3‐kinase (PI3K) pathway. *PIK3CA*, the gene encoding PI3K catalytic subunit p110α, is mutated in 20%‐40% of breast cancer patients. Several PI3K inhibitors have been developed and one, alpelisib, was recently approved for use in *PIK3CA*‐mutated, HR+, HER2‐negative advanced breast cancer. There are numerous types of assays and methods used in clinical studies to determine *PIK3CA* status in cancers. Additionally, there are several factors to consider for *PIK3CA* testing in clinical practice, including choice of assay, source of sample, and test timing. In this review, we discuss the use of *PIK3CA* as a biomarker to guide treatment decisions in patients with HR+, HER2‐negative advanced breast cancer, as well as practical considerations and recommendations for testing.

## Graphical abstract

*PIK3CA* may be used as a biomarker to guide treatment decisions in patients with HR+, HER2‐negative ABC. *PIK3CA* mutation may be assessed through either tissue or liquid biopsy using PCR‐ or NGS‐based assays.

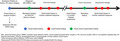

## 1 INTRODUCTION

Breast cancer is the most common cancer diagnosed in women and is the leading cause of cancer‐related deaths worldwide.^1^ In the United States, there will be an estimated 168 292 patients living with metastatic breast cancer in 2020.^2^ Approximately 71% of patients with breast cancer have hormone receptor‐positive (HR+), human epidermal growth factor receptor 2‐not‐amplified (HER2‐negative) disease, which is associated with a favorable short‐term prognosis.^3^ Current treatment options for HR+, HER2‐negative advanced breast cancer (ABC) include endocrine therapies (eg, tamoxifen and aromatase inhibitors), targeted therapies (eg, cyclin‐dependent kinase 4/6 [CDK4/6] inhibitors), and chemotherapy.^3^, ^4^

The HR+, HER2‐negative tumors are less sensitive to chemotherapy and, despite demonstrating improved clinical outcomes, patients eventually develop resistance to CDK4/6 inhibitors and endocrine therapies—especially in the advanced setting.^3^, ^4^ Other targeted therapies, such as phosphatidylinositol‐3‐kinase (PI3K) inhibitors, have been developed to overcome resistance to existing therapies.^4^, ^5^, ^6^

## 2 THE PI3K SIGNALING PATHWAY

The most frequently mutated signaling pathway in all breast cancers is the PI3K pathway.^7^ The PI3Ks are a family of lipid and serine/threonine kinases that integrate extracellular stimuli into intracellular signals to regulate various pathways that control several physiological functions including cellular proliferation, growth, survival, differentiation, and metabolism.^7^, ^8^, ^9^ Phosphatidylinositol‐3‐kinases are divided into 3 classes (I, II, III) based on substrate specificity and structure.^9^ Of the 3 classes, class I PI3Ks are the most extensively studied and most established as a cause of many cancer types.^9^, ^10^

Class I PI3Ks are subdivided into subclasses IA and IB in mammals based on their mode of regulation. Class IA PI3Ks are composed of a p110 catalytic subunit and a p85 inhibitory adaptor/regulatory subunit.^8^, ^9^ The genes *PIK3CA*,* PIK3CB*, and *PIK3CD* encode class IA catalytic isoforms p110α, p110β, and p110δ, respectively. Class IA and IB PI3Ks phosphorylate phosphatidylinositides (PtdIns(4,5)P2) in vivo, whereas class III PI3Ks phosphorylate PtdIns. Some evidence suggests that class II PI3Ks may also preferentially phosphorylate PtdIns in vivo.^9^

## 3 RATIONALE FOR TARGETING *PIK3CΑ* IN ABC

Constitutive activation of the PI3K pathway is commonly related to oncogenesis.^9^ Class I PI3Ks, which include PI3Kα, PI3Kβ, PI3Kγ, and PI3Kδ, are aberrantly activated in breast cancer.^7^ The most common mechanism leading to constitutive activation of the PI3K pathway is the somatic loss of phosphatase and tensin homologue (PTEN) by genetic or epigenetic alterations. Other mechanisms of PI3K pathway activation include the activation of receptor tyrosine kinases (RTKs) and PI3K isoform mutations, duplications, and/or overexpression.^9^

In breast cancer, *PIK3CA* mutations are the most frequent alterations in the PI3K pathway, with at least 80% occurring within the helical (E542K and E545K) and kinase (H1047R) domains of p110α.^11^ Mutations in *PIK3CA* have been reported in 20%‐40% of breast cancer cases, and their incidence also varies across different breast cancer molecular subtypes.^11^, ^12^, ^13^ The incidence of *PIK3CA* mutations has been estimated at 36%‐45% of HR+, HER2‐negative breast cancers; up to 40% of HER2‐positive breast cancers; and 9%‐14% of triple‐negative breast cancers.^12^, ^14^, ^15^

Regarding *PIK3CA* mutation as a prognostic factor, there are conflicting results on the association of *PIK3CA* mutations and breast cancer outcome.^16^ A pooled analysis showed that the presence of *PIK3CA* mutations is a negative prognostic factor (pooled overall survival [OS], disease‐free survival [DFS], and progression‐free survival [PFS]) in breast cancer.^13^ It has been suggested that *PIK3CA* may offer a differing prognostic role in early versus advanced or metastatic BC. Data from 10 319 early breast cancer patients with known *PIK3CA* genotype showed that *PIK3CA* mutations were associated with better invasive DFS, distant DFS, and OS. However, it should be noted that after adjusting for other factors including treatment, estrogen receptor/HER2 status, and tumor grade, the effect only remained significant for invasive DFS.^17^ In a recent subgroup analysis of the SAFIR02 study, patients with *PIK3CA‐*mutated, HR+, HER2‐negative metastatic breast cancer were found to be less sensitive to chemotherapy and presented with shorter survival than patients without *PIK3CA* alterations (OS hazard ratio [HR] 1.44; 95% CI, 1.02‐2.03; *P* = .039).^18^ In agreement with this, a systematic review of 12 studies conducted in HR+, HER2‐negative metastatic breast cancer reported worse prognosis in patients whose cancers had *PIK3CA* mutations compared with wild‐type when treated with non‐PI3K inhibitors. Notably, in patients treated with PI3K inhibitors, improved PFS was observed in the *PIK3CA*‐mutant cohort.^19^ However, other meta‐analyses have reported the presence of *PIK3CA* mutations to be associated with better relapse‐free survival.^20^ Overall, the prognostic significance of *PIK3CA* alterations in breast cancer remains inconclusive.

Mutations in *PIK3CA* may be found alongside *HER2* amplification and PTEN loss, which also enhance PI3K signaling activity.^20^ Phosphatase and tensin homologue protein loss is found in 35% of triple‐negative; 11% of HR+, HER2‐negative; 5% of HR+, HER2‐positive; and 6% of HR–, HER2‐positive breast cancers.^21^

Loss of PTEN is also thought to be associated with trastuzumab resistance. Preclinical studies have shown that *PIK3CA*‐activating mutations and/or PTEN loss diminishes the growth inhibitory effects of trastuzumab, and trastuzumab‐resistant cell lines have been shown to respond to a selective PI3K inhibitor. In clinical studies, PTEN loss and/or *PIK3CA*‐activating mutations have been associated with poor clinical outcomes and were not associated with outcomes related to treatment with trastuzumab. Despite worsening clinical outcomes, patients with PTEN loss still demonstrated benefit from trastuzumab treatment.^21^

## 4 PI3K INHIBITOR PHASE 3 CLINICAL TRIAL TESTING METHODS AND OUTCOMES

### 4.1 Pan‐PI3K inhibitor: Buparlisib

Buparlisib is an oral pan‐PI3K inhibitor that targets all four isoforms of class I PI3K. BELLE‐2 was a phase 3 trial that evaluated the safety and efficacy of buparlisib in combination with fulvestrant in postmenopausal women with aromatase inhibitor‐resistant HR+, HER2‐negative ABC. The status of PI3K in archival tumor tissue was determined during a 14‐day run‐in treatment phase using Sanger sequencing of the *PIK3CA* gene and immunohistochemistry of PTEN protein expression. Patients were classified into three categories: (a) PI3K pathway activated—if Sanger sequencing detected a mutation in *PIK3CA* exons 1, 7, 9, or 20, or if there was a loss of PTEN expression; (b) PI3K pathway non‐activated; and (c) PI3K pathway unknown—if the assessment was uninterpretable. Plasma samples for circulating tumor DNA (ctDNA) analysis were collected at study entry for all randomized patients. Analysis was done using beads, emulsification, amplification, and magnetics (BEAMing) based on a predefined panel of 15 *PIK3CA* mutations in exons 1, 7, 9, and 20 (Arg88Gln, Arg93Trp/Gln, Lys111Glu/Asn, Gly118Asp, Glu365Lys, Cys420Arg, Glu542Lys, Glu545Gly/Lys, Gln546Lys, and His1047Arg/Leu/Tyr) (Table 1).^5^, ^22^, ^23^, ^24^, ^25^, ^26^, ^27^ Patients treated with buparlisib (n = 576) had significantly improved median PFS (mPFS) compared with placebo (n = 571; 6.9 vs 5.0 months; HR 0.78; *P* = .00021). A significant improvement in mPFS was also observed in buparlisib‐treated patients with known PI3K status and patients with an activated PI3K pathway (Table 1). Exploratory analysis using ctDNA showed that patients with *PIK3CA* mutations (n = 200) had longer PFS when treated with buparlisib compared with placebo (Table 1). This was not observed in the ctDNA *PIK3CA* nonmutant cohort (n = 387).^22^

**Table 1:** *PIK3CA* mutation detection assay and sample types in phase 3 PI3K inhibitor trials

Investigational agent	Clinical trial	Method of detection	*PIK3CA* coverage	Type of sample	*PIK3CA*‐mutated				
					Group	n	mPFS, mo	HR (95% CI)	*P* value
Buparlisib	BELLE‐2^22^ (N = 1147)	Sanger sequencing	Exons 1, 7, 9, 20 (+PTEN IHC)	Archival tumor tissue	BUP	188^a^	6.8	0.76 (0.60‐0.97)	.014
					PBO	184^a^	4.0		
		BEAMing	15 point mutations on exons 1, 7, 9, 20	Plasma ctDNA at baseline	BUP	87	7.0	0.58 (0.41‐0.82)	.001
					PBO	113	3.2		
	BELLE‐3^23^ (N = 432)	BEAMing (Inostics)	8 point mutations on exons 9, 20	Plasma ctDNA at screening or at day 1 of cycle 1	BUP	100	4.2	0.46 (0.29‐0.73)	.00031
					PBO	35	1.6		
		PCR (Roche cobas assay)	17 point mutations on exons 7, 9, 20	New or archival tumor tissue	BUP	75	4.7	0.39 (0.23‐0.65)	<.0001
					PBO	34	1.4		
Taselisib	SANDPIPER^24^ (N = 631)	PCR (Roche cobas assay)	17 point mutations on exons 7, 9, 20	Not disclosed	TAS	340	7.4	0.70 (0.56‐0.89)	.0037
					PBO	176	5.4		
Alpelisib	SOLAR‐1^5^, ^25^, ^26^, ^27^ (N = 572)	PCR (QIAGEN therascreen *PIK3CA* RGQ)	11 point mutations on exons 7, 9, 20	New or archival tissue^b^	ALP	169	11.0	0.65 (0.50‐0.85)	.00065
					PBO	172	5.7		
		FoundationOne CDx 324‐gene NGS	All encoding exons	New or archival tissue^b^	ALP	121	11.0	0.59 (0.43‐0.82)	NS
					PBO	118	5.5		

Abbreviations: ALP, alpelisib; BEAMing, beads, emulsification, amplification, magnetics; BUP, buparlisib; CI, confidence interval; ctDNA, circulating tumor DNA; HR, hazard ratio; IHC, immunohistochemistry; mPFS, median progression‐free survival; NGS, next‐generation sequencing; NS, not specified; PBO, placebo; PCR, polymerase chain reaction; PI3K, phosphatidylinositol‐3‐kinase; *PIK3CA*, phosphatidylinositol‐3‐kinase p110α isoform; PTEN, phosphatase and tensin homologue; TAS, taselisib.

^a^ Patients were categorized as PI3K pathway‐activated (any mutation detected in *PIK3CA* or loss of PTEN expression).

^b^ New and archival tissues refer to samples taken ≤3 months and >3 months prior to randomization, respectively.

In BELLE‐2, the most common grade 3/4 adverse events (AEs) in the buparlisib group (n = 573) were elevated alanine aminotransferase (ALT; 25%), elevated aspartate aminotransferase (AST; 18%), hyperglycemia (15%), and rash (8%). Serious AEs occurred more frequently in the buparlisib group (23%) compared with placebo (16%). Mood disorders such as depression and anxiety were more common in the buparlisib group (buparlisib vs placebo: 26% vs 9% for depression and 22% vs 8% for anxiety). Suicidal ideation was noted in three patients in the buparlisib group and two in the placebo group, but no suicide attempts were reported. There were no noted occurrences of treatment‐related deaths.^22^

The phase 3 BELLE‐3 trial evaluated the safety and efficacy of buparlisib in combination with fulvestrant in postmenopausal women with HR+, HER2‐negative ABC who progressed on/after an aromatase inhibitor and resistant to endocrine therapy plus everolimus. Plasma samples collected at screening or at cycle 1, day 1 were analyzed for *PIK3CA* mutations status at exons 9 (Glu542Lys, Glu545Lys, Glu545Gly, Gln546Lys) and 20 (Met1043Ile, His1047Tyr, His1047Arg, His1047Leu) using the Inostics BEAMing assay for ctDNA analysis. Mutation status of *PIK3CA* was also analyzed via the Roche cobas^®^ *PIK3CA* assay that covers exons 7, 9, and 20, using new or archival primary or metastatic tumor sample (Table 1).^5^, ^22^, ^23^, ^24^, ^25^, ^26^, ^27^ Patients enrolled in the trial (N = 432) who were treated with buparlisib (n = 289) had significantly longer mPFS compared with placebo (n = 143; 3.9 vs 1.8 months; HR 0.67; *P* = .0003). Consistent with the results of BELLE‐2, patients with *PIK3CA* mutation by ctDNA treated with buparlisib (n = 100) also had significantly longer mPFS compared with placebo (Table 1).^23^

The safety profile of buparlisib plus fulvestrant in the BELLE‐3 trial was consistent with that of the BELLE‐2 trial. Serious AEs were reported in 22% and 16% of buparlisib and placebo groups, respectively. Adverse events that led to dose interruptions were more frequent in the buparlisib group than in the placebo group (36% vs 9%), as were dose reductions (31% vs 8%) or discontinuation (21% vs 5%). The most common reasons (all‐grade AEs) for permanent discontinuation of treatment with buparlisib were elevated ALT (6%), elevated AST (4%), and depression (2%). Suicidal ideation was noted in both treatment groups (2% vs 1%), and three suicide attempts were reported in the buparlisib group. Most on‐treatment deaths were due to metastatic breast cancer, but two were considered treatment‐related (cardiac failure [n = 1] in the buparlisib group and unknown reason [n = 1] in the placebo group).^23^

Efficacy results from BELLE‐2 and BELLE‐3 demonstrated that patients with *PIK3CA*‐mutant, HR+, HER2‐negative ABC benefited more from treatment with a PI3K inhibitor than patients without *PIK3CA* mutations. However, the toxicity associated with buparlisib did not support further development of the combination of buparlisib and fulvestrant.^22^, ^23^

### 4.2 Beta‐sparing PI3K inhibitor: Taselisib

The efficacy and safety of taselisib, a beta‐sparing^28^ PI3K inhibitor with enhanced activity in *PIK3CA* mutated breast cancer cell lines, were evaluated in the phase 3 SANDPIPER trial. *PIK3CA* mutation status was identified using centralized Roche cobas test (which detects the following mutations: R88Q, N345K, C420R, E542K, E545A/G/K/D, Q546K/R/E/L, M1043I, H1047L/R/Y, G1049R) (Table 1).^5^, ^22^, ^23^, ^24^, ^25^, ^26^, ^27^ A total of 631 postmenopausal patients with ER+, HER2‐negative ABC who had disease recurrence or progressed on aromatase inhibitor were included, 516 of whom were *PIK3CA*‐mutant. Taselisib in combination with fulvestrant improved investigator‐assessed mPFS compared with fulvestrant alone (Table 1). The most frequent AEs of any grade in the taselisib group were diarrhea (60%), hyperglycemia (40%), stomatitis (33%), nausea (34%), decreased appetite (26%), and rash (25%). Grade 3/4 AEs occurred in 50% of patients in the taselisib‐plus‐fulvestrant group and only 16% in the placebo‐plus‐fulvestrant group. Serious AEs were also more common in the taselisib group, occurring in 32% compared with 9% in the placebo group. Adverse events led to taselisib discontinuation, dose interruption, and dose reduction in 17%, 41%, and 37% of patients, respectively. Overall, investigators concluded that taselisib had a modest benefit but an unfavorable safety profile.^24^

### 4.3 Alpha‐specific PI3K inhibitor: Alpelisib

Alpelisib is an alpha‐specific PI3K inhibitor used in combination with fulvestrant for the treatment of postmenopausal women and men with HR+, HER2‐negative, *PIK3CA*‐mutated ABC. In the phase 3 SOLAR‐1 trial (NCT02437318), the safety and efficacy of alpelisib in combination with fulvestrant were evaluated in patients with HR+, HER2‐negative ABC who received prior endocrine therapy.^5^ Mutation status of *PIK3CA* in SOLAR‐1 was determined by using a validated clinical trial assay and was transitioned to QIAGEN therascreen^®^ *PIK3CA* RGQ polymerase chain reaction (PCR) kit to detect mutation hotspots in the C2 (C420R), helical (E542K, E545A, E545D, E545G, E545K, E545X, Q546E, Q546R, Q546X), and kinase domains (H1047L, H1047R, H1047X, H1047Y) of PI3K (exons 7, 9, and 20, respectively).^25^ Formalin‐fixed, paraffin‐embedded tumor samples were used for testing of samples obtained at initial diagnosis or at the most recent biopsy. In addition, ctDNA was obtained at baseline and analyzed by PCR to evaluate PFS by *PIK3CA* mutation status^26^ (Table 1).^5^, ^22^, ^23^, ^24^, ^25^, ^26^, ^27^

A total of 572 patients were included in the trial, 341 of whom had *PIK3CA*‐mutated ABC by tissue analyses, while 231 were included in the *PIK3CA*‐nonmutated cohort.^5^, ^26^ The primary endpoint of PFS in the *PIK3CA*‐mutant cohort was met. Progression‐free survival in the *PIK3CA*‐mutant cohort was significantly longer in the alpelisib‐plus‐fulvestrant group compared with the placebo‐plus‐fulvestrant group (11.0 vs 5.7 months; HR 0.65; *P* = .00065). Notably, the proof‐of‐concept criteria to determine if treatment benefit was obtained in the *PIK3CA*‐nonmutant cohort were not met.^5^

The most common AEs of any grade that occurred in the alpelisib‐plus‐fulvestrant group were hyperglycemia (64%), diarrhea (58%), nausea (45%), decreased appetite (36%), and rash (36%). Patients who discontinued treatment due to AEs with alpelisib and placebo were 25% and 4.2%, respectively. Hyperglycemia was the most common AE leading to alpelisib discontinuation, occurring in 6.3% of patients.^5^, ^29^ In patients treated with alpelisib, diabetic and prediabetic patients demonstrated greater mean increases in fasting plasma glucose from baseline than patients with normal glucose at baseline.^26^

In patients with *PIK3CA* mutations detected in ctDNA (n = 186), PFS was found to be significantly improved in alpelisib‐treated patients compared with placebo (10.9 vs 3.7 months; HR 0.55; *P* value not specified), consistent with the PFS results in *PIK3CA‐*mutant tissue samples.^26^ Retrospective testing (n = 415) using the FoundationOne CDx^®^ 324‐gene panel next‐generation sequencing (NGS) assay was done to further characterize *PIK3CA* alterations observed in patients from SOLAR‐1.^25^ The *PIK3CA* mutation status was generally consistent between PCR used at screening to classify patients and the NGS assay.^25^ Patients with altered *PIK3CA* status (as determined by NGS) showed a very similar improvement in PFS when alpelisib was added to fulvestrant treatment^27^ (Table 1).^5^, ^22^, ^23^, ^24^, ^25^, ^26^, ^27^ Overall, the results of SOLAR‐1 suggest that *PIK3CA* mutation status predicts response to alpelisib.

## 5 PRACTICAL CONSIDERATIONS FOR *PIK3CA* TESTING

In clinical practice, breast cancer patients may undergo core biopsy, providing adequate tissue for diagnosis and biomarker testing, with an option for subsequent excisional biopsy or mastectomy.^30^ As such, there is typically sufficient tissue available. However, it can be challenging to implement biomarker testing across samples in clinical trials—especially when there is a limited amount of tumor sample.^31^ As a recent example, 27% of patients in the SOLAR‐1 trial did not undergo NGS testing due to insufficient quantity or quality of the tissue samples.^25^

Another important issue is tissue preservation and preparation, which can affect the sample quality. Delays in fixation may lead to degradation of RNA and protein, which may cause alteration of immunohistochemical results; however, the American Society of Clinical Oncology—College of American Pathologists (ASCO‐CAP) guideline recommendations for biomarker testing have practically eliminated this as an issue with requirements for immediate fixation for a duration of 6 hours and not to exceed 72 hours and mandatory recording of the times in the surgical pathology report.^32^, ^33^, ^34^, ^35^, ^36^, ^37^ Other factors that should be taken into consideration include the type of test required, concordance between different tests, and testing centers. Communication between the clinicians and pathologists is essential to determine the type of sample, method of processing, and tests required.^31^

Tumor heterogeneity and evolution should also be considered. For some types of alterations, increased rates of cell proliferation and mutation combined with genomic instability lead to significant genetic variations and intratumor heterogeneity; however, such heterogeneity for standard breast cancer biomarkers is observed in only approximately 1% of primary breast cancer biopsies.^31^, ^38^ Incidence of tumor heterogeneity within metastases may also occur more frequently relative to primary sites—including occurrence of *ESR1* mutations in metastases that lead to acquired resistance to antiestrogen therapy (such as aromatase inhibitors).^39^, ^40^, ^41^ Hence, it has been suggested that biomarker testing should be done in both the primary and metastatic/recurrent lesions to account for tumor heterogeneity.^42^ However, it may be difficult to obtain metastatic specimens of adequate quality since core biopsy specimens tend to be smaller, which can result in insufficient sample for molecular analysis and lead to impurities due to stromal contamination. Re‐biopsies may not always be feasible due to the locations of the metastatic site, and the procedure may be associated with increased morbidity. Furthermore, a comparative genomic analysis done comparing the primary tumors and matched metastatic lesions from 23 patients with breast cancer showed that mutational profiles were concordant except for one patient.^43^ Thus, overall, the choice of targeted therapy may change relatively infrequently when the metastatic lesion is profiled versus the primary lesion.

Although there are multiple types of assays available for screening, two of the most commonly used assays in clinical trials for breast cancer are PCR and NGS. Polymerase chain reaction assays are low‐cost, sensitive, rapid tests that can detect specific target mutations but do not provide a comprehensive analysis of genes being investigated. Next‐generation sequencing assays are highly sensitive and also have the ability to assay all mutations in the genome, including rare sequences and complex mutations.^31^, ^44^ In the retrospective NGS analysis of *PIK3CA* mutation in SOLAR‐1, NGS was able to detect 60 different mutations across multiple exons and five copy number variations, in contrast to point mutations across three exons by PCR. In addition, out of 175 patients assigned to the nonmutant cohort by PCR, NGS testing revealed that 28 (16%) of these patients had a *PIK3CA* alteration. Also, NGS detected multiple *PIK3CA* mutations in 44 patients, six of whom had no detected mutations by PCR.^25^ Despite increased sensitivity and wider coverage, NGS does have some drawbacks, including higher cost and longer turnaround time.^31^ Currently, two assays have been approved by the US Food and Drug Administration for *PIK3CA* testing in advanced breast cancer. The therascreen *PIK3CA* RGQ PCR Kit (QIAGEN GmbH) is a companion diagnostic approved for use with alpelisib in combination with fulvestrant for the treatment of HR+, HER2‐negative *PIK3CA*‐mutated advanced or metastatic breast cancer (following progression on or after an endocrine‐based regimen).^6^, ^21^, ^45^ The assay is capable of detecting 11 frequently observed mutations in the *PIK3CA* gene (exon 7: C420R; exon 9: E542K, E545A, E545D [1635G>T only], E545G, E545K, Q546E, Q546R; and exon 20: H1047L, H1047R, H1047Y).^45^, ^46^ The FoundationOne^®^ CDx assay is also approved as a companion diagnostic for detection of those mutations.^47^

Tissue‐based testing is considered standard for diagnosis and treatment selection because of its widespread use and the availability of substantial evidence to support its use in early and advanced cancers.^48^, ^49^, ^50^ Molecular characterization of tumor tissue samples remains the current gold standard of personalized medicine.^49^ However, tissue‐based testing is invasive, may be associated with complications of biopsy, and may not be feasible for all patients (patient too ill, or site not accessible to biopsy).^48^, ^51^ In recent years focus has shifted to DNA‐based biomarkers. In contrast to tissue‐based testing, ctDNA assays are minimally invasive, making them ideal for serial monitoring.^52^ Compared with protein‐based biomarkers, ctDNA has a greater dynamic range and shorter half‐life (<2.5 hours), which allows it to be a more sensitive indicator of tumor progression and treatment response. Genetic alterations leading to treatment resistance may also be identified using ctDNA. Studies have demonstrated that ctDNA can be used to detect early recurrence in asymptomatic breast cancer patients who have undergone curative surgery. However, detection of early recurrence via biomarker analysis has not yet been shown to improve patient outcomes. Further, disease recurrence may occur without an increase in biomarker levels. Studies also suggest that in addition to identifying mechanisms of acquired resistance, these assays can be used to monitor treatment response in patients with ABC.^52^ Serial plasma specimens collected from 30 women with ABC receiving systemic therapy were analyzed for ctDNA to monitor tumor burden using assays that were designed to detect somatic genomic alterations (including point mutations in *PIK3CA* and *TP53*) previously identified using targeted or whole genome sequencing. In addition, cancer antigen 15‐3 (CA 15‐3) and circulating tumor cells (CTC) were measured at the same time points.^53^ CA 15‐3 is a serum biomarker that can be used to monitor treatment and prognosis of patients with breast cancer, but it has low sensitivity and specificity.^52^, ^53^ ctDNA, CA 15‐3, and CTC were then compared to radiographic imaging, which is the standard for noninvasive monitoring of treatment response in ABC. ctDNA was identified in 97% (29/30) of samples, while CA 15‐3 and CTC were detected in 78% (21/27) and 87% (26/30) of women, respectively. ctDNA was found to have a greater correlation with changes in tumor burden than CA 15‐3 and CTC and also provided the earliest measure of treatment response in 53% (10/19) of women.^53^ Studies are also mostly retrospective; hence, further clinical validity and utility studies using ctDNA‐based monitoring are still needed.^48^ ctDNA assays are also relatively expensive, labor intensive, and not widely available. Examples of ctDNA biomarkers in breast cancer include *ESR1*, *PIK3CA*, and *TP53*.^52^ For the alpelisib PCR companion diagnostic, ctDNA derived from plasma of breast cancer patients or genomic DNA extracted from tumor tissue can be used.^46^

## 6 RECOMMENDED *PIK3CA* TESTING PRACTICES

The results of phase 3 trials have been informative and support the utility of *PIK3CA* mutation testing to guide treatment decisions in patients with HR+, HER2‐negative ABC. These results also demonstrate the importance of stratification based on genetic testing at randomization (particularly the BELLE‐3 trial).^23^

Overall, the reverse transcriptase PCR‐based companion diagnostic for alpelisib is likely sufficient for most clinicians’ needs. However, the assay is limited to the detection of specific mutations and does not account for less commonly observed mutations in *PIK3CA*, *PIK3CA* copy number alterations, or alterations in other genes relevant to PI3K signaling activity such as *PTEN*.^46^ When adequate resources are available, clinicians may want to consider supplemental testing to detect a wider array of PI3K pathway aberrations.

**Figure 1:**
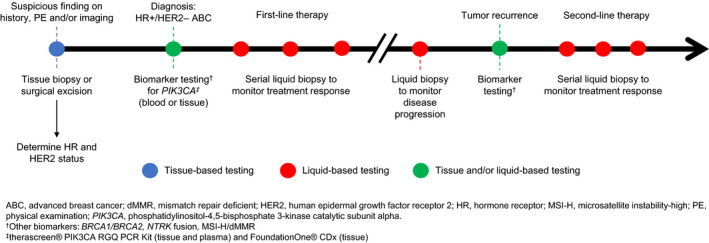
Potential timepoints for tissue and liquid biopsy testing in HR+/HER2–ABC. ABC, advanced breast cancer; dMMR, mismatch repair deficient; HER2, human epidermal growth factor receptor 2; HR, hormone receptor; MSI‐H, microsatellite instability‐high; PE, physical examination; *PIK3CA*, phosphatidylinositol‐4,5‐bisphosphate 3‐kinase catalytic subunit alpha. ^†^Otherbiomarkers:*BRCA1/BRCA2*,* NTRK* fusion, MSI‐H/dMMR.^30^ ^‡^ *therascreen* ^®^ PIK3CA RGQ PCR Kit (tissue and plasma) and FoundationOne^®^ CDx(tissue).^47^, ^55^

As discussed above, there is substantial agreement between primary and metastatic lesions, so either source would be acceptable for informing treatment decisions.^43^ To address potential concerns with sample quality and degradation as well as potential tumor evolution over time, it is recommended to coordinate efforts and use fresh samples for testing when possible.^31^ In terms of timing, it is recommended to test for *PIK3CA* mutation at diagnosis of metastatic disease to see if the patient is suitable for alpelisib treatment (Figure 1).

Although liquid biopsies are increasingly utilized, caution is warranted. Most patients have concordant tissue and liquid biopsy results, but liquid biopsies have a lower sensitivity and discordant results can occur. In most cases of discordance, the tissue sample is positive while the liquid biopsy does not detect a mutation. Hence, it is recommended that a reflex tumor tissue biopsy is done when no mutation is detected via liquid biopsy. When liquid biopsy does not detect a mutation, this may mean either the absence of the mutation in the tumor or a low amount of ctDNA in the sample. When a mutation is detected in liquid biopsy but not in tissue, it may be due to either temporal (archival tumor specimen) or spatial (subclonal mutation) heterogeneity.^48^ A tissue sample only represents a single tumor region (one primary or metastatic site) and provides a snapshot of the time the sample was taken. Thus, it would not be reflective of the entire tumor molecular landscape.^48^, ^50^ In contrast, liquid biopsy may capture tumor sample ctDNA arising from all metastatic sites and can be done sequentially.^48^, ^50^ Patients with tumor‐undetected and liquid biopsy‐positive results are less likely to benefit from targeted therapy due to tumor heterogeneity. Lastly, an assay error may also occur that could give false‐negative tissue genotyping or a false‐positive ctDNA genotyping. It is also important to consider that there is no consensus regarding the timing of biomarker analysis. For early‐stage cancer, the clinical utility of using ctDNA for diagnosis is limited because mutations are generally detected at a lower rate than in advanced cancers. In advanced cancers, literature supports the use of ctDNA for treatment selection during disease progression rather than when the patient is still responding to prior therapy. When a tumor is responding to therapy, ctDNA levels may be low, thus decreasing odds of mutation detection (if present).^48^

Although NGS‐based assays can detect genomic variations that may respond to targeted therapy, clinical utility in breast cancer outside of clinical trials has yet to be established.^48^, ^54^ A retrospective study was done in 44 metastatic breast cancer patients who underwent targeted NGS testing (FoundationOne) using the Illumina HiSeq 2000 platform. Sixteen patients were ER+, 4 were HER2‐positive, and 24 were triple‐negative. Almost all patients (n = 42, 95%) had actionable mutations, but only 55% (n = 23) of those with actionable mutations initiated targeted therapy, which suggests a lack of access to targeted therapies with approved indications and a lack of enrolling clinical trials.^54^ However, as suggested by results from the SOLAR‐1 study, a number of patients were initially classified as not having altered *PIK3CA* by reverse transcriptase PCR analysis who were later reclassified after NGS testing. Further studies are needed to determine the incidence of rare *PIK3CA* mutations and to understand the impact of these mutations on clinical outcomes in the HR+, HER2‐negative ABC population.

## 7 SUMMARY AND CONCLUDING REMARKS

In a disease wherein molecularly targeted therapies have been limited to HER2 and estrogen receptor, PI3K inhibitors are a new class for which *PIK3CA* mutations predict response. Although CDK4/6 inhibitor‐based regimens have become the standard of care for advanced HR+, HER2‐negative ABC, the only biomarker that appears to predict response for that class of drugs is *ESR1*, which is not the molecular target. Although further studies are needed to establish the prognostic significance of *PIK3CA* mutations in advanced breast cancer, *PIK3CA* mutations predict a favorable response to PI3K inhibitor treatment.

When testing for *PIK3CA* mutations in ABC, various factors should be considered by pathologists and oncologists, including choice of assay, sample availability, sample collection and preparation, time of screening, and tumor heterogeneity and evolution.^14^, ^16^, ^31^, ^44^ Liquid biopsy technology is evolving but requires further evaluation. As previously noted, reflex tumor tissue biopsy is recommended for negative results due to considerable rates of discordance with tumor genotyping.^48^ Next‐generation sequencing‐based assays can be considered but may have limited utility or availability in most clinical settings depending on expertise and reimbursement considerations. However, as additional molecular targets are identified in breast cancer, it is possible that NGS may become the standard of care for testing similar to what has occurred for stage IV lung cancer. With the approval of alpelisib along with therascreen *PIK3CA* RGQ PCR Kit (QIAGEN GmbH) and FoundationOne^®^ CDx as companion diagnostics, *PIK3CA* testing practices may become standardized and widespread, and real‐world use should provide more information about the incidence and pathology of various *PIK3CA* alterations in HR+, HER2‐negative ABC.

## CONFLICT OF INTEREST

The authors have disclosed the following potential conflicts of interest: Toppmeyer: Spouse employed by Merck; Press: Research grants to author's institution: Cepheid, Eli Lilly & Company, Novartis Pharmaceuticals, F. Hoffmann‐La Roche Ltd, Zymeworks; Consulting or advisory role with honoraria: Karyopharm Therapeutics, Puma Biotechnology, Biocartis, Eli Lilly & Company, Novartis Pharmaceuticals, F. Hoffmann‐La Roche Ltd; Expert testimony: Amgen; Travel, accommodations or expenses: Novartis.

## AUTHOR CONTRIBUTIONS

Both authors contributed to the manuscript equally: conceptualization, writing ‐ original draft, and writing ‐ review and editing.

## Data Availability

Data sharing is not applicable to this article as no new data were created or analyzed in this study.
